# Enhancing operational efficiency through overall equipment efficiency optimization and Kaizen initiatives

**DOI:** 10.1371/journal.pone.0320761

**Published:** 2025-05-09

**Authors:** Abu M. Fuad, Nusra Akter Takia, Hasin Ahmad Zafir, Omar Farrok

**Affiliations:** 1 Department of Electrical and Electronic Engineering, Ahsanullah University of Science and Technology, Dhaka, Bangladesh; 2 Department of Electrical and Electronic Engineering, University of Scholars, Dhaka, Bangladesh; 3 Department of Mechanical and Production Engineering, Islamic University of Technology, Gazipur, Bangladesh; Manipal Academy of Higher Education, INDIA

## Abstract

This case study aims to investigate the causes of low efficiency in a carton production line by calculating Overall Equipment Effectiveness (OEE). A typical carton factory has been chosen for this study, which has two production lines, namely finished goods and corrugated boards. The define, measure, analyze, improve, and control approach is applied while implementing Six Sigma principles such as Kaizen, fishbone diagrams, and 5W+1H as its systematic procedure. The analysis involves estimating four machines’ average OEE across 12 shifts. A cost-effective method is applied to resolve the problems that cause the lines to be less efficient. By applying the proposed method, the OEE becomes more efficient by 29% for finished goods and 9% for corrugated boards. Value stream mapping has been used to track the improvements. It is found that emphasizing higher OEE values enhances operational performance, leading to better efficiency, power usage, cycle time, and equipment repair.

## 1. Introduction

Over the years, the packaging industry has gone through revolutionary changes. The carton-packing sector has been playing a major role in this sector. The demand for effective, ecological, and secure packaging solutions has increased as the world becomes more linked. The market for folding cartons is projected to increase from USD 162.50 billion in 2022 to USD 226.10 billion by 2029. The significance of cartons goes beyond simple confinement. They play a crucial role throughout the entire product lifetime through their functions in product protection [1], logistical effectiveness, sustainability [2], and preserving product integrity [3]. The context of the global commerce ecosystem highlights this crucial character even more. Numerous factors influence global commerce, which has a significant impact on countries’ economies. Recent adversities, most notably the COVID-19 epidemic, have brought attention to the need for trustworthy packaging in industries like e-commerce [4].

The carton sector is facing difficulties that could affect its efficiency and sustainability, despite its obvious importance. This article takes a close look at the current state of the carton packaging business and points out its operational problems. It further explores ideas and methods that have been put into action to make production more efficient. With a focus on a carton plant that embodies more general industry pain factors, the study takes a two-pronged approach. First, it analyzes and comprehends lengthy cycle times, insufficient efficiency, and equipment problems. Second, it investigates the effectiveness of Kaizen concepts and tools, such as Value Stream Mapping (VSM), as viable remedies [5]. Operational efficiency is enhanced through overall equipment effectiveness optimization and kaizen initiatives. The proposed research methodology incorporating DMAIC (an acronym of a six-sigma tool; the first letters of the words Define, Measure, Analyze, Improve, and Control) is explained in section 3 (methodology). This case study’s contributions can be pointed out as follows.

- An assessment of the major roadblocks of carton factories, including long cycle times, inefficiencies, and equipment problems.
- An analysis of applying the concepts of Kaizen that can solve problems and promote advancements in the sector.
- Exploration of Value Stream Mapping’s role in identifying and bridging efficiency gaps.
- Strategic advice based on research insights that emphasize improvement over time and higher Overall Equipment Efficiency (OEE) values.

This study tries to answer the following question: How can operational performance be enhanced in carton production lines by incorporating OEE optimization and Kaizen initiatives within the DMAIC framework? More precisely, how can these strategies address inefficiencies, congestion, and maintenance challenges?

## 2. Literature review

### 2.1. Evolution of carton packaging

The late 19th century saw the invention of folding cartons, which provided adaptable and affordable packaging, and marked a turning point in the history of the carton packaging business [6]. Carton motion during the packaging process has been investigated mathematically, with a focus on online vectors, screw theory, and graph theory.

The industry has embraced recycled paperboard and renewable fibers in response to consumer demand for sustainability and environmental concerns [2]. Advances in printing technology, especially digital printing, have made packaging design more flexible by enabling high-quality graphics, personalization, and shorter print runs. Carton packaging has undergone a revolution thanks to technologies like RFID tags and sensors, which have improved functionality and customer involvement [7]. Tamper-evident measures such as holographic seals and tear strips have been incorporated to guarantee the integrity of the product [3]. The industry has adjusted to the growth of e-commerce by creating packaging specifically for online retail, maximizing dimensions for effective shipping, and improving product protection during transit [8]

### 2.2. Advances in carton packaging

The carton packaging sector has grown significantly over the last two to three decades due to changes in customer tastes, technical innovation, and sustainability initiatives. Automation, digital printing, and computer-aided design (CAD) software are examples of technological developments that have improved manufacturing efficiency and design flexibility [8]. The industry has embraced water-based inks, bio-based coatings, and recycled paperboard in response to consumer demand for environmentally friendly choices. To improve operational efficiency and optimize supply chain processes, lean manufacturing principles and continuous improvement approaches have been implemented [9].

Ensuring consistent product quality, safety, and environmental responsibility is of utmost importance. Lean manufacturing principles and continuous improvement methodologies have been embraced to enhance operational efficiency and optimize supply chain processes. The industry has focused on reducing waste and streamlining operations through lean practices [9]. Quality control and compliance with safety standards are paramount in the carton industry. Quality assurance certifications such as ISO 9001 and ISO14001 are widely adopted to ensure consistent product quality, safety, and environmental responsibility. Overall, these developments and trends have shaped the carton industry, fostering growth, sustainability, customization, operational efficiency, and adherence to quality and safety standards.

Scholars and practitioners have focused on what should be measured and how to evaluate cost-effectively in the carton sector, drawing a lot of attention to performance measurement [10] Businesses prioritize productivity optimization [11] and accessibility of industrial facilities [12]to stay competitive. Industrial productivity losses are divided into three categories using the Overall Equipment Effectiveness (OEE) framework: availability, performance, and quality. An OEE of 85% or more is frequently linked to world-class status [13, 14]. The carton industry is shaped by these practices and trends, which promote growth, sustainability, personalization, operational efficiency, and adherence to quality and safety regulations.

### 2.3. Addressing limitations in the carton industry

The carton sector faces a few issues that affect quality and productivity, such as poor maintenance procedures, aging equipment, ineffective changeover procedures, unskilled staff, ineffective quality control methods, and unproductive production scheduling. Aging machinery is less efficient and requires more frequent repairs, while inadequate maintenance causes malfunctions and unscheduled downtime [15]. Production delays and higher downtime are caused by inefficient changeovers [16]. Inadequate staff proficiency leads to mistakes and longer downtime. According to [17] inefficient quality control results in waste, rework, damaged cartons, and lower customer satisfaction. Consequently [18], ineffective production planning results in underutilization and decreased productivity, while ineffective coordination creates delays and bottlenecks. Process optimization techniques such as Kaizen, VSM, Six Sigma, and the DMAIC approach provide organized means of tackling these issues and improving overall performance.

### 2.4. Six Sigma tools to be used

#### 2.4.1. DMAIC.

Defects Per Million Opportunities (DPMO) is the Six Sigma failure metric that displays failure per million opportunities. 3.4 DPMO is the prime goal of Six Sigma control. The number of errors that will arise from doing an action a million times is indicated by DPMO [19]. The Six Sigma team in completing specific projects to reach the Six Sigma level needs to be guided by the 5 phases of the DMAIC [2]. DMAIC is the process used to identify the root causes of problems and implement sustainable solutions that improve processes and performance [20]. This process is regularly used in applications of Lean and Six Sigma [21,22]. “The five steps of DMAIC correspond to each letter in the acronym and stand for 1) Define - identify the problem and opportunity for improvement, 2) Measure - evaluate the current state, 3) Analyze - measure the current state and determine cause-and-effect relationships, 4) Improve - implement changes to correct the problem and capitalize on the opportunity, and 5) Control - ensure the changes that are implemented are sustained” [23–25].

#### 2.4.2. Brainstorming.

Brainstorming is a popular technique for coming up with unique ideas and solutions that have been shown to promote a variety of viewpoints, participation, and creativity. Research demonstrates its ability to promote dissenting opinions and enable concept exploration in an unrestricted and judgment-free setting. Nonetheless, studies indicate that using structured protocols and encouraging favourable group dynamics might improve the efficiency and results of brainstorming sessions. A good example may be seen in [26] where participants jotted down ideas before moving from a group environment to private areas to further explore the ideas they had selected. This study includes brainstorming sessions with pertinent staff members to create a fishbone diagram that shows the main sources of waste and bottlenecks in the carton production process.

#### 2.4.3. Why-why analysis.

The Why-Why analysis, sometimes referred to as the five whys technique, is a problem-solving methodology that uses a sequence of “why” questions to identify the root cause of a problem. It is extensively used in a variety of industries for decision-making, problem-solving, and quality improvement. Difficulties include the requirement for skilled facilitators and constraints when managing intricate, multifaceted issues. To improve the approach and investigate how it may be combined with other strategies, more study is required. Why-Why analysis has been used well in case studies, such as the ones examined by [27], to pinpoint underlying causes, especially when it comes to off-centre drilling problems. This technique efficiently identifies the underlying reasons for recognized problems and helps eliminate identified causes through critical few-cause analysis. The Why-Why analysis is used in this study to classify products into the 4M (Man, Material, Machine, Method) and Environment categories to find defects in the manufacture of carton sheets. The Why-Why table focuses on defect causes in particular- rough edges.

#### 2.4.4. Fishbone diagram.

Often called a fishbone diagram, the Ishikawa diagram is a visual aid for root cause analysis and issue analysis. The diagram organizes potential causes into groupings such as people, processes, equipment, environment, and materials to provide a thorough knowledge of complicated issues. Research highlights its importance in process and quality enhancement, encouraging methodical thinking, collaboration, and efficient problem-solving. Bottlenecks and waste causes were found in recent research on the process flow of the carton industry [28]. These findings were portrayed in a fishbone diagram that was created using the results of brainstorming sessions. The way the diagram was arranged helped to suggest process modifications that addressed the Krepek effect [29]

#### 2.4.5. Kaizen.

As studies have repeatedly shown, kaizen, which is Japanese for “continuous improvement,” is a key idea in lean management and process optimization. Its advantages include increased productivity, decreased waste, better quality, and increased employee engagement. Long-term increases in productivity and competitiveness can be substantial when a culture of continuous learning and small changes is established using Kaizen approaches [30,31] provides a detailed account of the conventional method of implementing Kaizen in an industrial setting, along with an understanding of how it interacts with other components of Lean production. Furthermore, a case study on the application of Kaizen, as reported in [32] shows promising outcomes, confirming the usefulness of Kaizen in the automated and commercial sectors. While the advantages of kaizen are well recognized, putting it into practice is not always easy. Leadership buy-in and a growth mindset are critical to a successful Kaizen initiative, according to Brunet and New [47]. In these areas that are reluctant to change, the results might not be adequate without them. There has also been a lack of effort to tailor Kaizen’s ideas to industry-specific challenges, such as unreliable machinery and variable output. In their warning against a cookie cutter approach, Bessant et al. [48] argue that businesses like carton manufacturers need individualized solutions.

#### 2.4.6. 5W+1H table.

The who, what, when, where, why, and how questions make up the 5W+1H approach, a potent framework for information collecting and analysis. It permits a thorough investigation of a subject or circumstance, ensuring that all crucial factors are considered. Applying the 5W+1H strategy has been found to improve problem-solving, decision-making, and communication because it encourages a thorough investigation of pertinent elements and facilitates the development of a holistic knowledge of difficult challenges. The 5W+1H method is presented by [27] to get recommendations on different proposed improvements from three types of defects that occur during the production of invitation papers. The paper, however, failed to meet the proposed Six Sigma criterion after implementing this method. To find out the main topics of improvement and choose the optimum topics that only satisfy the questions of Who, where, why, what when, and how the 5W+1H method is used in this paper. This is summarized in Table 1.

**Table 1:** Analysis and problem-solving methodologies for the carton industry.

Topic	Discussion summary	Impact on research	References
Brainstorming	A good tool for discussion	Analysis of problem	[26]
DMAIC	Methodological Importance	Determine the issue and find a solution.	[23–25]
Why-Why	Can identify vital few causes	Analysis of problem	[27]
Fishbone	Identifies the root cause	Analysis of root-cause	[16,18,29]
5W+1H	Finds improvement paths	Examination of possible improvement paths	[27]
OEE	Identify faulty machines	Calculation of improvement ventures	[33]
Kaizen	Continuous improvement	Definition of improvements and their applications	[31]
Value Stream Mapping	Maps the information and process flow well	Unified approach towards explaining the problem	[34]

### 2.5 Research gap

Notwithstanding considerable progress in carton packaging technology and processes, a major disparity persists in the practical use of integrated process optimization tools such as OEE and Kaizen in actual manufacturing environments. Current research mostly concentrates on theoretical frameworks or discrete enhancements, lacking a thorough implementation of methodical techniques like the DMAIC framework in conjunction with tools like 5W+1H and fishbone diagrams. In the context of carton manufacturing lines, it is unknown how the techniques affect quantifiable results such as efficiency, power use, cycle time, and upkeep of equipment. Through exploring the efficiency issues of a standard carton plant with two separate production lines (finished items and corrugated boards), this research fills up these gaps. This case study demonstrates a quantifiable improvement in OEE values—29% for finished goods and 9% for corrugated boards—by applying Six Sigma principles methodically and using an economical problem-resolution approach. The result serves to close the gap between theoretical research and practical, data-driven solutions. The outcomes demonstrate the importance of utilizing increased OEE for long-term operational enhancements and applications to the industry.

## 3. Methodology

**Fig 1:**
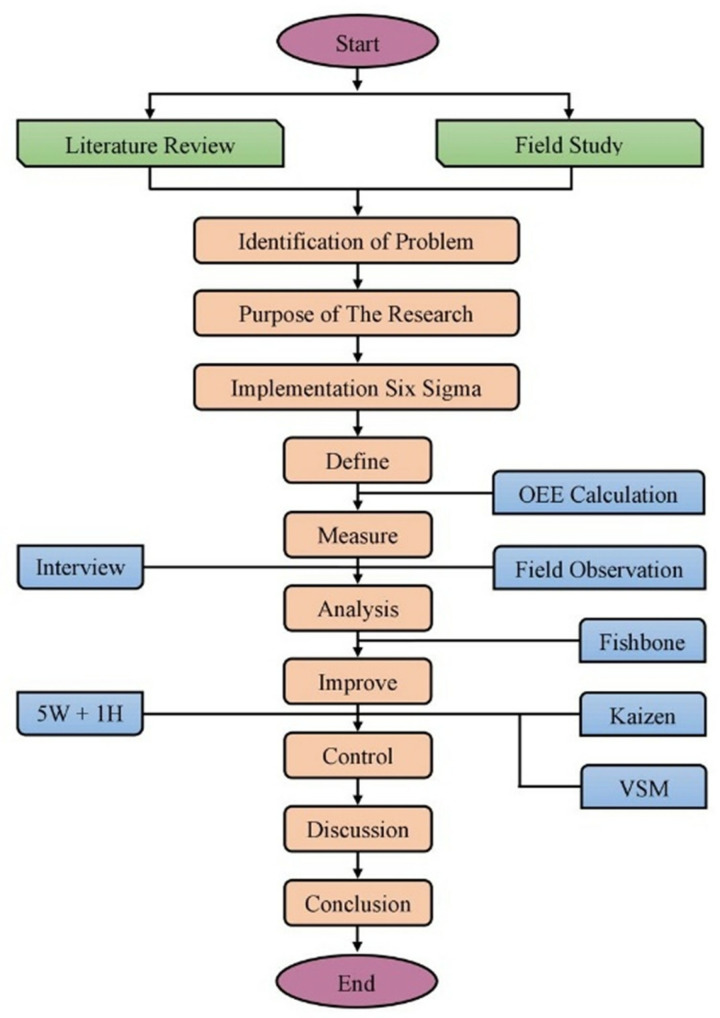
Proposed research methodology incorporating DMAIC.

The issues faced in the carton manufacturing industry were identified and addressed using a systematic manner (DMAIC), which was the technique used throughout this study. It requires the selection and application of multiple six-sigma tools throughout each stage of DMAIC. Implementing Six Sigma concepts involves adopting a structured approach to improve processes, reduce defects, and enhance overall organizational performance. Six Sigma is a data-driven methodology that focuses on minimizing variations and achieving standard quality levels. It follows a structured approach known as DMAIC, which stands for define, measure, analyze, improve, and control. By following the DMAIC approach, organizations can systematically identify process issues, make data-driven improvements, and establish control measures to ensure sustained results. It provides a structured framework for problem-solving and continuous improvement in an industry as shown in Fig 1.

### 3.1 Data collection and analysis

#### 3.1.1. Define phase (D).

**Fig 2:**
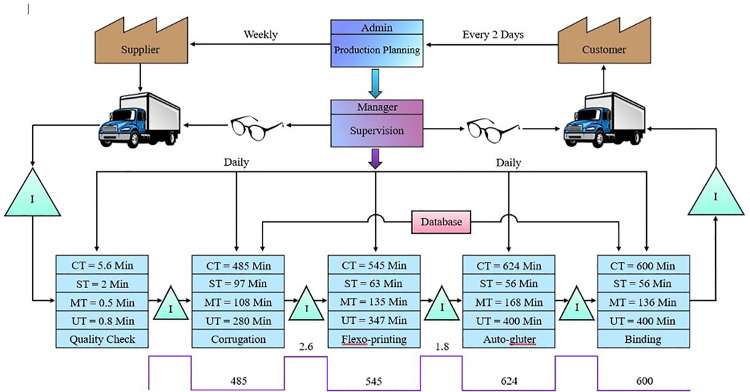
VSM of the production process.

The production process of carton sheets has been mapped by using value stream mapping as sketched in Fig 2. It is a series of steps that raise a product’s worth to its anticipated level. VSM refers to the process of mapping the needs and data in a value stream that encompasses production, suppliers, and distribution to the customer. Value Stream Mapping is used in this situation to save cycle time. A case study of the use of VSM in the crankshaft production process is examined in [35] customers, process stages and metrics, inventory, supplier material flows, information, physical flows, lead time, and takt time are the main components of this VSM. In Fig 2, VSM of the whole production process is depicted with an average takt time curve given for the whole operation.

Extensive research has been done on the value and importance of VSM which is an efficient method to map out the critical value-adding points of manufacturing [36].

The OEE metric is the fundamental component of the total productive maintenance manufacturing improvement methodology according to [37] which is founded on three connected ideas:

- Maximizing equipment efficiency
- Autonomous maintenance by operators
- Small-group exercises

As a result, OEE may be viewed as combining the administration, upkeep, and operation of manufacturing resources and equipment [38] OEE is effective for single pieces of equipment [39–41] and highly automated processes [39]. Research shows that [42] OEE can determine which machine performs the poorest and where to focus on the machines that process work independently.

#### 3.1.2 OEE Calculation.

The proportion of truly productive time to anticipated production time is known as OEE. OEE is measured by taking the product of availability, performance, and quality using Equation (1) [43]. The availability ratio is a measurement of the amount of time a process or machine is used to the amount of time it was intended to run for using Equation (2).


 OEE =  Availability × Performance ×  Quality (1)

Availability considers Downtime (loss), and is calculated as


$$\text{ Availability}=\;\frac{\text{(Planned operating time – Downtime) }}{\text{Planned operating time}}$$ (2)

Performance is the net ratio of actual output to target output. This is the output by reducing downtime loss from the planned operating time to the target output, which refers to the machine runtime in a planned operating time designed machine output. The performance of a machine is used to determine the efficiency of the equipment that produces output compared to its designed potential. It displays the equipment’s operating speed as a percentage of its intended (ideal) speed. It considers speed losses. Performance is improved by decreasing speed losses, lowering machine idling time, and removing unplanned downtime using Equation (3).


$$\text{Performance }=\frac{\text{Actual output}}{\text{Target output}}$$ (3)

Here, Target output = (Designed machine Output × Planned operating time)

Quality is improved by eliminating starting losses which can be considered corrugation margin loss (some of the cardboard has to be left out during the initial cut), rework, and quality flaws. The remaining time is “fully productive time” and is considered a loss. Quality is an essential indicator for OEE since it shows what percentage of finished goods are defect-free. To what extent does the manufacturing system reduce faults and guarantee this metric quantifies product dependability? The quality can be expressed as the ratio of excellent goods to total products divided by the number of defective parts as follows.


$$\text{Quality }=\frac{\text{(Total produced parts – Defects parts) }}{\text{Total produced parts}}$$ (4)

**Fig 3:**
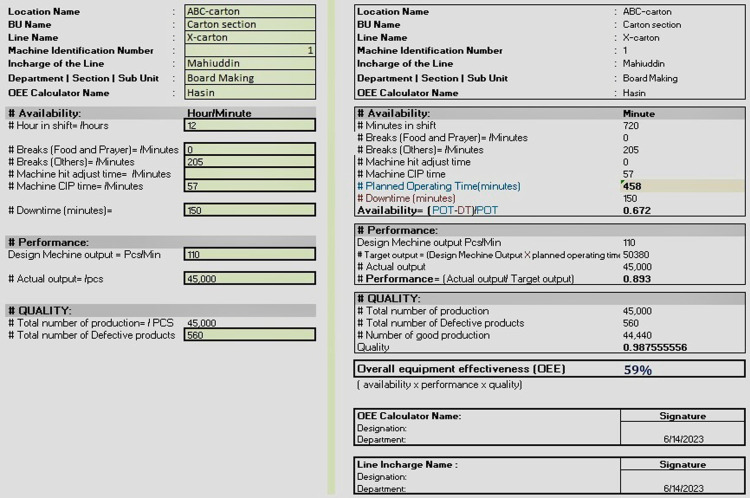
OEE calculation with developed calculator for a carton factory: (a) inputs for calculation (b) report generation.

According to [33], the dominant character for calculating OEE is the performance among three parameters. As a result, the cost of their downtime or inactivity increases significantly. Similar problems have been found while preparing a result. Apart from that, the machine remains off due to the unavailability of raw material, machine breakdown, and item change process loss which are stated in Tables 2–4. Evaluation of the extent of various production losses is required for the implementation of total productive maintenance to optimize resource allocation and activity direction. Therefore, regular maintenance of the plant’s machinery is crucial for Breakdown Maintenance [43]. The OEE calculation report for the X-carton Line at the ABC-carton location in the carton section reveals certain issues that need to be addressed in the carton factory, as shown in Fig 3. The identified problems are as follows.

**Table 2:** OEE of auto corrugation machine for 12 shifts.

Parameters (minutes)	No. of shifts	1	2	3	4	5	6	7	8	9	10	11	12
OEE (%)	63	63	64	68	78	62	63	70	66	73	73	70
No demand													
Item change process loss		73	54	10	10		22	30	10	12	40	23	
Scheduled maintenance		10	20	30	30		0	10	10	10	10	10	
Machine breakdown				0	0		15				50		
Food and prayer		60	60	60	60		60	60	60	60	60	60	60
Paper torn		25.2	34.02	65	68		80	70	80	75	14	80	
Total downtime (hours)		2.8	2.8	2.75	2.8	0	2.95	2.83	2.67	2.62	2.9	2.89	1

**Table 3:** OEE of flexo printing machine for 12 shifts.

Parameters (minutes)	No. of shifts	1	2	3	4	5	6	7	8	9	10	11	12
OEE (%)	65	69	73	56	63	70	66	73	68	66	66	65
No demand		240	0	720									
Item change process loss		70	75		120	40	10	12	40	85	60	100	90
Scheduled maintenance							10	10	10	30		7	12
Machine breakdown		40							50				
Raw material shortage						68					40		
Food and prayer		60	60	60	60	60	60	60	60	60	60	60	60
Paper torn							80	75	14				
Total downtime (hours)		2.83	2.25	1	3	2.8	2. 67	2.62	2.9	2.92	2.67	2.78	2.7

**Table 4:** OEE of auto-glutter machine for 12 shifts.

Parameters (minutes)	No. of shifts	1	2	3	4	5	6	7	8	9	10	11	12
OEE (%)	65	69	73	62	64	65	68	69	75	72	59	67
No demand		240	0	720				40					
Item change process loss		70	75		120	40	117	40		119	110	85	60
Scheduled maintenance								20				30	
Machine breakdown		40											
Raw material shortage						68							40
Food and prayer		60	60	60	60	60	60	60	60	60	60	60	60
Paper torn													
Total downtime (hours)		2.83	2.25	1	3	2.8	2.95	2.67	1	2.98	2.83	2.92	2.67

*Availability issue:* The availability of the X-carton Line is calculated to be 0.672, indicating that the machine is operational for approximately 67.2% of the Planned Operating Time (POT) of 458 minutes. A significant amount of downtime, totaling AU150 minutes, affects availability. The reasons behind this downtime, such as breaks (others) or machine-specific issues, should be investigated and resolved to improve availability further.

*Performance issue:* The design machine output is set at 110 pieces per minute, resulting in a target output of 50,380 pieces for the shift (POT multiplied by the design machine output). The actual output recorded is 45,000 pieces, which indicates a performance level of 89.3% (actual output divided by target output). Although the performance is relatively high, further analysis is needed to identify any factors that may be limiting the machine’s output and explore opportunities for improvement.

*Quality issue:* The total number of productions during the shift is recorded as 45,000 pieces, out of which 560 pieces are defective. This suggests a quality level of 98.8% (number of good productions divided by the total number of productions). While the quality level is relatively high, efforts should be made to reduce the number of defective products and further enhance the overall quality performance.

Overall, the carton factory faces challenges in availability, performance, and quality aspects. The identified issues in these areas should be thoroughly investigated and addressed to improve the OEE. By focusing on minimizing downtime, optimizing performance, and enhancing quality control measures, the carton factory can achieve higher OEE (%) and ensure more efficient operations.

#### 3.1.3. Measure phase (M).

**Fig 4:**
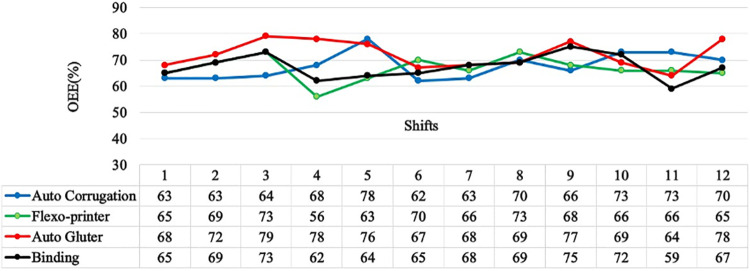
OEE plot of different machines over 12 shifts.

OEE is an effective approach for locating hidden industrial inefficiencies and losses. A crucial step towards world-class lean manufacturing for businesses of all sizes and sectors is tracking OEE scores and using them to drive changes in manufacturing processes. The OEE of the four machines was determined for 12 shifts using the data that was gathered (as stated in Tables 2–5) and the data for 12 shifts were plotted in Fig 4.

**Table 5:** OEE of binding machine for 12 shifts.

Parameters (minutes)	No. of shifts	1	2	3	4	5	6	7	8	9	10	11	12
OEE (%)	68	72	79	78	76	67	68	69	77	69	64	78
No demand			0					40					
Item change process loss		20	56	10	30	60	60	40	40	50	50	60	
Scheduled maintenance				20		30	30	20	20	20	20	20	
Power shutdown				20									
Machine breakdown											20	30	
Food and prayer		120	120	60	60	60	60	60	60	60	60	60	60
Paper torn					30								
Total downtime (hours)		2.33	2.9	1.8	2	2.5	2.5	2.67	2	2.16	2.5	2.83	1

## 4. Analysis and results of Kaizen implementation

### 4.1. Analysis phase (A)

**Fig 5:**
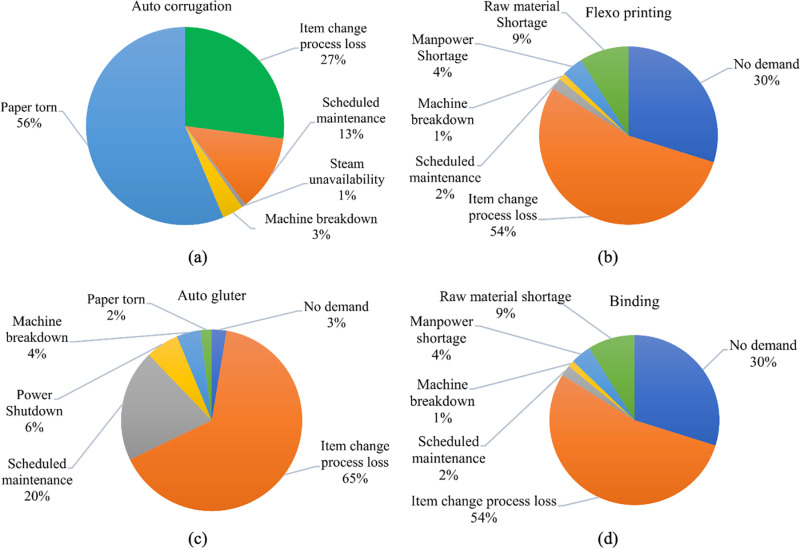
Pie chart representing downtime for (a) auto corrugation machine, (b) flexo printer machine, (c) auto glutter machine, and (d) binding machine.

OEE can help identify the root causes by analyzing the three main parameters of performance, quality and availability. Analyzing this allows for the identification of the underlying issues [44]. Table 6 summarizes the average overall equipment effectiveness for four machines based on information from Tables 2–5. Their quality rate, performance efficiency, and availability are all revealed by this analysis. The average OEE of auto-corrugation, flexo printer, auto glutter, and binding machines is 62.5%, 59%, 62.5, and 58.5%, respectively. The average availability of auto-corrugation, flexo printer, auto glutter, and binding machines is 0.7115, 0.7005, 0.777, and 0.66, respectively. The data from Fig 5 shows that paper tear and item change processes are the most frequent causes of downtime in these machines. The three primary causes of downtime for auto corrugation machines, as indicated in Fig 5(a), are item change processes, paper tearing, and planned maintenance. An average value of 62.5% for OEE demonstrates that the equipment efficiency is good enough given that these operations make up a significant portion of the system. The equipment undergoes heavy usage and needs several maintenance procedures. The auto-corrugation machine’s average OEE value ranges around 63% across the 12 analyzed shifts. The data indicates that there was no demand during any of the shifts, resulting in no production during those periods (shifts). The “item change process loss” parameter represents the time wasted during item change processes. This loss varied from 10–73 minutes, with different durations for each shift.

**Table 6:** Availability, performance, and quality of the machines for different shifts.

Parameters	Availability	Performance	Quality	Availability	Performance	Quality
For 1, 4, 19, and 11 shifts			For 6, 13, 13, and 18 shifts		
Auto-corrugation	0.773	0.825	0.985	0.65	0.88	0.984
Shifts	1			6		
OEE (%)	63			62		
Flexo printing	0.648	0.873	0.985	0.753	0.843	0.973
Shifts	4			13		
OEE (%)	56			62		
Auto glutter	0.777	0.825	0.985	0.777	0.806	0.984
Shifts	19			13		
OEE (%)	63			62		
Binding	0.648	0.873	0.985	0.672	0.893	0.987
Shifts	11			18		
OEE (%)	59			85		

Additionally, the machine experienced scheduled maintenance, ranging from 10–30 minutes during specific shifts. There were no reported instances of power shutdown, steam unavailability, manpower shortage, or raw material shortage affecting the examined shifts, an auto-corrugation machine. The “paper torn” attribute indicates that the machine had downtime from 14–80 minutes throughout various shifts as a result of paper tearing accidents. The auto-corrugation machine had downtime overall because of paper ripping, planned maintenance, and item change process loss. The item change process loss and the lack of steam are the main causes of downtime in the flexo printer shown in Fig 5(b). Since this machine is the first step in the printing process, matching the colour to the customer’s specifications takes some time, which results in downtime. The OEE values associated with auto glutter machines ranged around 68% in the auto glutter machine, which was generally acceptable. The primary cause of downtime in the auto glutter machine and binding machine shown in Fig 5(c) and (d) is item change process loss as well. Since this procedure is the final portion of the output, inspection is routinely performed to guarantee accuracy in the output.

#### 4.1.1. Why-why analysis.

Defective items due to paper torn or rough edges, dented have an impact on downtime in a manufacturing process. The main factors causing defects are made in Table 7. In this table, the cause of the defects in the defective products is divided into several categories, namely 4M (man, material, machine, method) and environment. The causes of downtime due to defects in carton sheet production are determined using participation and observation studies and brainstorming with managers, supervisors, and relevant employees.

**Table 7:** Why-Why table for identification of causes of defects.

Why	Why	Why	Why	Why
Defect	Rough edges	Man	The machine’s indications are not understood by the operators.	The operator lacks experience
		Material	Poor quality of raw material	Not the standard brand
		Machine	Irregular cutting problem	There is no routine maintenance
		Method	A problem in cutting and sewing	Operators do not follow the effective way
		Environment	Moist	Inappropriate room temperature and humidity

#### 4.1.2. Fishbone diagram.

**Fig 6:**
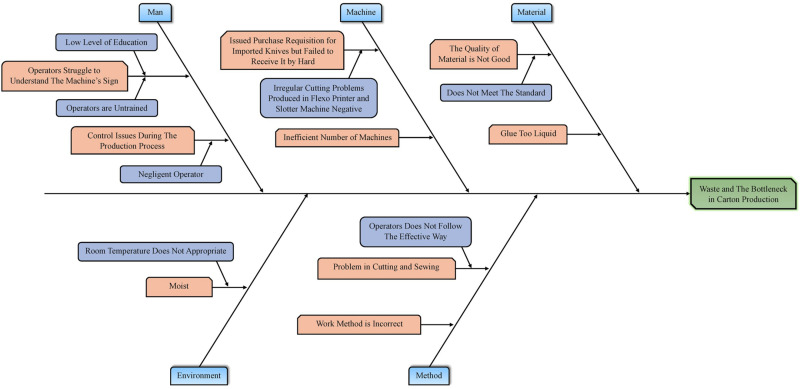
Proposed fishbone diagram of waste and the bottleneck of carton production.

A fishbone diagram is used to identify the source of the issue that results in defects during carton sheet production. The reasons of waste and bottlenecks in carton manufacture are shown in Fig 6, which is a cause-and-effect (fishbone) diagram. The diagram accentuates numerous critical issues contributing to waste and carton production obstacles. The expressions “Operators struggle to understand the machine’s sign” and “Operators are untrained” indicate deficiencies in competence and communication. Difficulties like “Irregular cutting problems in the Flexo Printer and Slotter Machine” and “Inefficient number of machines” indicate equipment inefficiencies. Concerns with the substance, such as “The quality of the material is not good” or “Glue too liquid,” highlight the use of low-grade components as shown in Fig 6.

### 4.2. Improvement phase (I)

The 5W + 1H approach is used to make changes in the enhancement part. By using previously created training data, this technique determines whether a phrase has the 5W + 1H (what, who, where, when, why, and/or how) or not. “5W + 1H” containing sentences will be chosen as summary sentences. The action taken to improve using the 5W + 1H strategy is listed in Table 8.

**Table 8:** Comprehensive improvement initiatives with the 5W + 1H method.

What	Where	Why	Who	When	How
Increase in worker’s proficiency with machinery	Production floor	Therefore, operators can reduce defect	Head of production and head of training	Done for one to two months	Provide rigorous routine training and selection
Improvement of the room environment	Production floor	Employer health and safety, the performance of machines	Maintenance team, health and safety department	During the production process	Set up the exhaust fan at the corner of the production floor
Improvement in the cutting process	Production floor	Hence, operators can reduce rough edges	Quality control manager	During the production process	Surface grind to get better cutting results
Supervision of operator	Production floor	To improve discipline	Supervisor	During the production process	By supervising the performance of each operator in their fields
Improvement of gluing material	Raw materials warehouse	To get the desired production results	Quality control manager	Every raw material entered	Make observations before using the glue
Security of operator	Production floor	To prevent accidents and maintain the stamina of workers	Head of production	During the production process	Educating operators on the significance of PPE
Increase in the number of employees	Production floor	To focus operators working on each part thus reducing the defect	Assistant manager	When the machine is idle (No operator)	Recruitment
Inspection of raw material	Raw materials warehouse	Ensuring good quality raw materials	Quality control manager	During raw material intake	Improvement through Kaizen

**Note:** *PPE means Personal Protective Equipment*

Based on the results of the fishbone study, the increase in faults and downtime is mostly due to a lack of machine expertise among operators. This lack of understanding leads to less precise work practices and frequent operational concerns. Therefore, the proposed action to overcome this is to conduct regular training for the operators and assess the results of training. Furthermore, the performance of machine operators will be evaluated to improve operating performance in the future.

#### 4.2.1. Kaizen implementation.

Kaizen, the Japanese word for continuous improvement, calls for ongoing communication between peers and supervisors (horizontal) and between employees and managers (vertical). Kaizen is characterized by attention to detail and workable solutions promoting employee input. Efficient management of human resources with a focus on group responsibility for issue solutions is a strategic objective. Smoother carton edges and an annual cost savings of $7200 were achieved by using Kaizen concepts on the Flexo printer and slotter machine.

**Fig 7:**
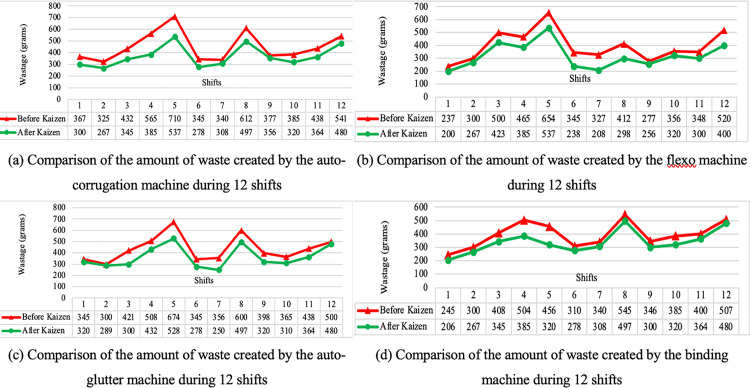
Comparison of wastage following the implementation of Kaizen across the four machines: (a) waste of auto-corrugation machine, (b) waste of flexo machine, (c) waste of auto glutter machine, (d) waste of binding machine.

Every year the knife was changed twice a year for 3 flexo-printers. The knives were repaired as part of the Kaizen initiative, positive feedback was received economically, whereas it saved, 1200$(per knife) × 6 = 7,200$. The comparison of the before and after shows improved environmental impact, safety, and quality. After implementing Kaizen, the auto corrugation machine results a significant decrease in waste in the carton sector over all 12 shifts, with the highest improvement occurring during shift 5, Flexo printer and slotter machine went through the best improvement during shift 7. The auto-glutter machine and binding machines experienced the most improvement during shifts 3 and 4, respectively, improving overall competitiveness. Fig 7 illustrates a comparison of waste following the implementation of Kaizen across the four machines, where different machines with 12 hours have been explored by waste. Comparisons between before and after Kaizen implementation are shown in Table 9.

**Table 9:** Comparison of before and after Kaizen implementation.

Subject		Process/ Project	
Flexo printer and Slotter machine		Printing labels and cutting in a batch	
Initial Condition (What was the problem?)		Solution Description (What was implemented, changed, or improved?)	
Irregular cutting problems produced negative feedback		Surface grind to get better cutting results	
Issued PR (Purchase Requisition) for imported knives, but failed to receive it by hand		Modify existing cutter	
Before applying Kaizen		After applying Kaizen	
5-ply (rough edges)	Customer Complaints	5-ply (smooth edges)	Customer Satisfaction
3-ply (rough edges)	Customer Complaints	3-ply (smooth edges)	Customer Satisfaction
Dull Knife	Slow lead time	Modified Knife	Fast lead time

#### 4.2.2. Work study improvement.

This study focuses on the problem identified in the carton factory: the high cost associated with printing travel card pages. To address the high-cost issue, the work method improvement focused on the reduction of pages for printing travel cards. Kaizen helps identify and eliminate waste, inefficiencies, and non-value-added activities in the production process as shown in Table 10.

**Table 10:** Comparison of printing pages before and after applying Kaizen.

Subject	Process/project
Travel Card	Printing pages
Initial condition (what was the problem?)	Solution description (What was implemented, changed, or improved?)
Too much initial cost	Reduction of pages
Before applying Kaizen (Include pictures, diagrams, etc.)	After applying Kaizen (Include pictures, diagrams, etc.)
2 Pages (Per page $ 0.03, with print)	1 Page, average saving of $174 per year whereas monthly saving is $14.5 (approximately)

In this Kaizen template, the subject of improvement is the printing of travel card pages in the carton and section department/division. The initial problem was that the cost associated with printing, the travel card pages was too high. To address this issue, the solution implemented was the reduction of pages. Before applying Kaizen, the process involved printing 2 pages per travel card. However, after applying Kaizen, the number of pages was reduced to 1 per travel card. This change resulted in a cost reduction, as each page cost $0.03 (with print). On average, the implementation of this improvement led to a total annual savings of $174. Additionally, the monthly savings were approximately $14.5.

In summary, the work method improvement in the carton factory’s travel card printing process resulted in substantial cost savings, improved quality, environmental benefits, enhanced delivery, increased end-user responsiveness, improved efficiency, boosted morale, and reduced waste. By implementing this Kaizen initiative, the carton factory achieved continuous improvement in its operations, leading to overall performance enhancement.

### 4.3. Control phase (C)

Control is the stage when process performance is monitored, and issues are prevented from reoccurring. Control charts are the often-employed tools. The following are the general purposes of control charts,

- Help reduce variability.
- Always monitor performance.
- Permits rejection to be avoided through the repair procedure.

A lack of long-term data collection prevents the study from including various control charts, thus the OEE standard values were compared with calculated values to determine the position of the metrics after application of Kaizen. The following standard values for the OEE component metrics were given in [45],

- Availability of more than 90%.
- Performance effectiveness of at least 95%.
- More than 99% quality.

**Fig 8:**
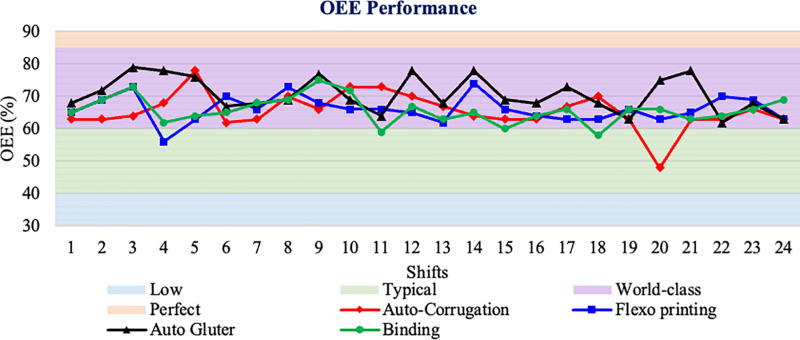
Comparison of OEE performance in different machine parameters such as auto-corrugation, flexo printing, auto glutter, and blinding, against world-class standards (10/12/23).

The average performance efficiency was 68%, the average performance availability rate was 80%, and the average quality value was 99%, which are all below the optimal OEE levels suggested in [33]. Estimates that such availability, performance, and quality levels would lead to an OEE of about 85% as estimated in [45]. However, some researchers have shown that OEE can range from higher than 50% [46]between 30% and 80% and between 60% and 75% [33]. An OEE over 85% is typically regarded as a standard for world-class performance. It’s crucial to remember that different businesses and industries may have varying standards and expectations. Based on the provided data, comparing these values to the benchmark of 85% for world-class OEE, it can be seen that the machines in the dataset after improvement activities are achieving world-class OEE levels shown in Fig 8. However, it’s important to consider the specific context, industry norms, and goals set by the company when evaluating OEE performance. The provided data can serve as a starting point for identifying areas of improvement and implementing strategies to enhance OEE and overall operational efficiency.

**Fig 9:**
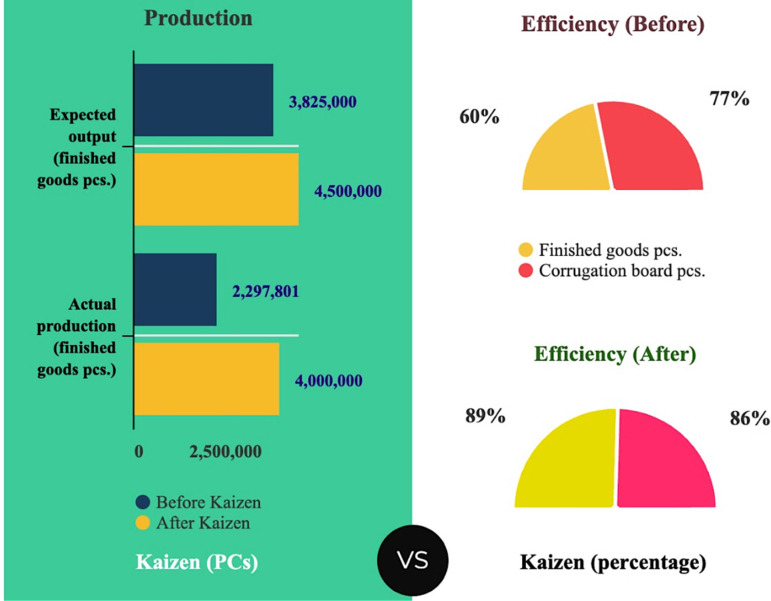
Comparison of expected and actual production in ABC carton line before and after applying Kaizen.

For finished goods, the anticipated output ranges from 3,825,000–4,500,000 pieces, while the actual production falls between 2,297,801–4,000,000 pieces. Similarly, for corrugation boards, the expected output is between 3,750,000 and 4,500,000 pieces, whereas actual production ranges from 2,898,696–3,875,000 pieces. Before Kaizen’s implementation, the carton line’s (finished goods) efficiency was 60%, operating at 60% of its full potential with an expected output of 3,825,000 pieces. Actual production fell short at 2,297,801 pieces. Similarly, for the carton line (corrugation board), efficiency was 77%, with an expected output of 3,750,000 pieces and an actual output of 2,898,696 pieces. Post-Kaizen, there was a substantial improvement in efficiency, reaching 89% for finished goods and 86% for corrugation board. Expected outputs were 4,500,000 pieces for both lines, with actual outputs of 3,875,000 and 4,000,000 pieces, respectively. These improvements stem from Kaizen initiatives involving process optimization, employee training, equipment repair, and addressing quality issues. The lines yielded 3,875,000 and 4,000,000 pieces, respectively, compared to the expected 4,500,000 pieces for both lines. Kaizen initiatives about process optimization, personnel training, equipment repair, and quality concerns resolution are the source of these gains. A comparison between the expected and actual production in the carton line under consideration before and after applying Kaizen is illustrated in Fig 9.

## 5. Discussion

This case study conducted in a large packaging industry provides a good context on how different Six Sigma tools can be implemented in an industry focusing on a large number of end products. Six Sigma tools have already been used widely in the production sectors of such industries. However, the application of OEE within the DMAIC methodology has not been tested in the existing literature [23–25].

In this work, the main source of inefficiencies of two production lines has been figured out following the implementation of OEE. This type of multi-production line configuration is common across many industries that produce more than one skew of an end product [20]. Furthermore, the respective 29% and 9% growth seen in the finished products and corrugation board manufacturing lines show the advantageous impact of making gradual modifications. The increase in efficiencies happened due to a small change in the form of repairing a cutting blade and decreasing the paper count in a flier. This is a user-friendly example of Kaizen principle. Previous studies have highlighted the problem of availability identified in this case study along with the problems of worse edge quality over time [22]. This phenomenon produced ill responses from customers. Bad customer reviews heavily affect industries which are in the growing stage and thrive on good reviews to get more future customers. A well-implemented OEE calculation can pinpoint the parts where inefficiencies take place very easily [23,25].

The discoveries of this research are broadly applicable to various situations because of the universal approaches used and the shared difficulties tackled. The research employs universally acknowledged concepts including DMAIC, Kaizen, and OEE, which are applicable across other sectors outside carton manufacturing. This study addresses prevalent problems like as downtime, equipment inefficiencies, and quality control throughout industries, including automotive, electronics, and food packaging. The measurable enhancements—29% in OEE for finished items and 9% for corrugated boards—illustrate the efficacy of these solutions, which may be customized to meet industry-specific requirements. The research highlights cost-efficient and data-informed strategies, including operator training and equipment optimization, assuring relevance for resource-limited companies. These results provide a reproducible framework for operational excellence in many industrial settings.

Future research should investigate the long-term viability of the enhancements and examine the incorporation of modern technologies, such as business 4.0 projects, in the carton business. Furthermore, doing comparative research across different organizations and nations may provide valuable insights into the flexibility and adjustment of the strategies. The research limits encompass factors such as the specific industry context, sampling quantity and participation, timescale, data availability, and value, as well as ecology and sustainability aspects. If the study largely concentrated on local or regional surroundings, it might have overlooked the wider ramifications of transnational supply chain phenomena on the carton business.

The industry situation after the implementation of Kaizen and other tools referenced throughout the study is reflected in the standards of OEE being more in line with the world-class standard. Attention must be drawn to the amount of savings achieved with Six Sigma, which was expected according to [17]

## 6. Conclusion

OEE and its practical implementation for managing and monitoring machine advancements are the subject of this case study. The implementation of Kaizen activities in the carton plant has led to substantial improvements across several aspects of the production process. The reduction in the cycle times of critical devices has resulted in enhanced productivity and accelerated task fulfillment. The enhanced functionality of the line signifies improved utilization of resources and decreased waste. Consequently, this has led to financial savings, improved energy efficiency, and enhanced product excellence. It has strategically positioned itself to achieve higher levels of productivity and industry competitiveness. The study’s findings provide many different industries with significant ideas and proposals for improving their overall performance and effectiveness. By optimizing OEE and effectively managing machine improvements as proposed in this paper, companies can reduce waste and achieve improved operational efficiency and productivity gains. This work adds to the existing information about operational excellence approaches in the manufacturing sector from an academic perspective. This framework enables further examination and confirmation in many industrial settings. The effective implementation of Kaizen enhanced Overall Equipment Efficiency and the utilization of fishbone diagrams in the carton sector demonstrate the concrete advantages of engaging in continuous improvement endeavors for professionals in the field. This research emphasizes the significance of cultivating a culture that promotes ongoing growth and adaptability in response to changing market requirements.

## Supporting information

S1 Data NPT of X-Carton. (XLSX)

S2 Data OEE Calculation of X-Carton. (XLSX)

S3 Data Result of NPT X-Carton. (XLSX)
